# Retrieving orbital angular momentum distribution of light with plasmonic vortex lens

**DOI:** 10.1038/srep27265

**Published:** 2016-06-03

**Authors:** Hailong Zhou, Jianji Dong, Jihua Zhang, Xinliang Zhang

**Affiliations:** 1 Wuhan National Laboratory for Optoelectronics, School of Optical and Electronic Information, Huazhong University of Science and Technology, Wuhan, 430074, China

## Abstract

We utilize a plasmonic vortex lens (PVL) to retrieve the orbital angular momentum (OAM) distribution of light. The OAM modes are coupled to the surface plasmon polaritons (SPPs) in the form of various Bessel functions respectively. By decomposing the interference pattern of SPPs into these Bessel functions, we can retrieve the relative amplitude and the relative phase of input OAM modes simultaneously. Our scheme shows advantage in integration and can measure hybrid OAM states by one measurement.

Surface plasmon polaritons (SPPs) are surface electromagnetic waves originating from collective oscillations of free electrons at a metal/dielectric interface. It has attracted lots of research interests because of its intriguing properties such as field localization and enhancement. The SPPs have been widely used in a variety of interesting applications, such as plasmon focusing^1^,^2^,^3^,^4^,^5^,^6^,^7^,^8^,^9^,^10^, directional plasmon coupling^11^,^12^ and so on. Plasmonic vortex lens (PVL), which enables to couple the input light to SPPs in the form of plasmonic vortex, is one of the most representative branches of SPPs. The plasmonic vortex refers to an optical vortex of plasmonic waves with a dark spot and phase singularity at its center. The PVL has lots of potential applications in recent years because of the tight focusing and nanoscale confinement of SPPs. Especially, the PVL can convert the right or left circularly polarized (RCP or LCP) light into a sharp focal spot without phase singularity. These features make the PVL can be applied in nanofocusing^13^,^14^,^15^ and circular polarization analyzer^16^,^17^,^18^,^19^,^20^,^21^. In addition, the PVL can couple the orbital angular momentum (OAM) into the form of various Bessel functions^6^,^7^,^8^,^9^,^10^,^11^,^12^,^13^,^14^,^15^,^16^,^17^,^18^,^19^,^20^,^21^,^22^ and it was employed to measure the OAM of light^7^. Light beams carrying OAM are associated with an azimuthal phase structure 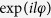, where *φ* is the angular coordinate and *l* is the azimuthal index, defining the topological charge (TC) of the OAM beams^23^. These beams have an OAM of 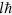 per photon (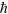 is Planck’s constant *h* divided by 2*π*). These OAM beams have been widely used in a variety of interesting applications, such as in quantum information^24^,^25^ and optical communication^26^,^27^. Apparently, the capability of retrieving different OAM modes is essential in OAM-based optical system. In fact, the OAM modes can be mapped to different Bessel functions through reasonably designing the PVL structure and this feature shows potential for retrieving the OAM distribution of light from the intensity patterns of SPPs.

In this work, we put forward an integrated scheme to retrieve the OAM distribution of light with PVL. The OAM modes are coupled to SPPs in the form of various Bessel functions respectively. By decomposing the interference pattern of SPPs into these Bessel functions, we can retrieve the relative amplitude and the relative phase of input OAM modes. Our scheme can measure the amplitude and phase distribution of OAM modes simultaneously. It offers an integrated scheme to measure hybrid OAM states by one measurement.

## Results

### Principle

**Figure 1:**
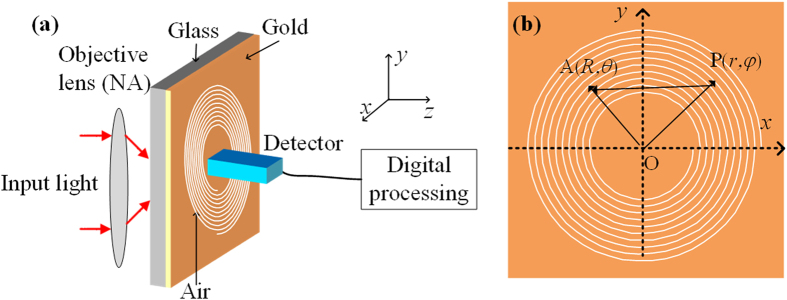
(**a**) The structure of PVL and the measurement system. (**b**) Top view of the PVL.

Figure 1(a) illustrates the structure of PVL and the measurement system. A metallic (gold) film with spiral air-slit is deposited on the glass substrate. The SPPs can be excited at dielectric/metal interface when the input light focused by an objective lens normally illuminates the metallic film from the glass substrate side. A detector is used to record the intensity distribution of SPPs and then the OAM distribution of input light is analyzed by digital processing. Figure 1(b) shows the top view of the proposed PVL structure. In the cylindrical coordinates, the geometry of *N*-fold spiral slits (*P*) can be described as


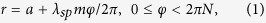


where 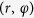 are the position of *P* in two-dimensional polar coordinates, 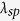 is the wavelength of the SPPs, *a* is the initial radius and *m* defines the geometrical charge of the PVL.

When an RCP or LCP OAM mode, whose initial amplitude is 1 and initial phase is 0, illuminates the metallic film, the plasmonic field at a generic point 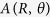 is expressed as a Bessel function^2^,^9^


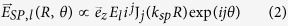


where 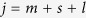 is the charge of plasmonic vortex, s = 1, *or* −1 represents the LCP or RCP light respectively, *l* is the TC of input OAM mode, 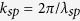 is the wavenumber of the SPPs. 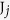 is *j*-th order Bessel function of the first kind, 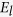 is a constant that relates to the loss. After the geometrical charge and the handedness of circularly polarized light are designated, the plasmonic field distributions are fixed in the form of various Bessel functions when different OAM modes shine the metallic film. That is to say, the plasmonic field distribution is the linear superposition of these Bessel functions when hybrid OAM modes with different amplitude and phase shine simultaneously. Therefore it is possible to determine the relative amplitude and relative phase of the OAM components via decomposing the intensity distribution of plasmonic field into these Bessel functions.

Now we assume that the complex amplitudes of the input OAM modes are 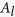, where 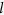 is the TC of corresponding OAM mode. From Eq. (2), we can derive that the superposition of plasmonic field is


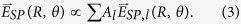


Because the intensity distribution of SPPs (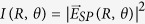) can be measured, the rest of key issue is to solve the problem in the following:


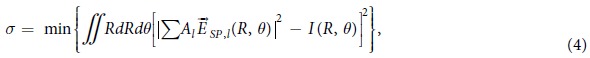


where Function min(X) returns the smallest value of X, *I* represents the measured intensity of interference pattern, and 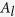 are the complex amplitudes to be retrieved. We assume that the effective detecting plane is a square with 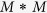 pixels, Eq. (4) can be revised to


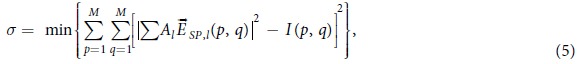


where *p* and *q* are the pixel values of the detecting plane. This non-linear least squares problem can be easily solved by employing the Levenberg-Marquardt algorithm^28^. The Levenberg–Marquardt algorithm is an iterative procedure and converges to the global minimum only if the initial guess is already somewhat close to the final solution. In this scheme, all the initial guesses for the complex amplitudes are set as 1. It should be noted that this scheme can only get the relative phase of OAM modes, that is to say, if adding a common phase to all 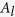, Eq. (5) still holds up. In addition, the coefficients 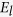 in Eq. (5) are unknown, so what we retrieve are selected as the parameters 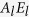 in the Levenberg-Marquardt algorithm. To evaluate the performance of the algorithm, we define a parameter of relative squared residual (RSR) in the following to determine whether the guess is close to the final solution:


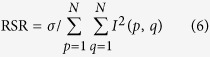


### Simulations

In fact, the number of spiral will not affect the plasmonic field distribution around the center except the coupling efficiency. Considering the device size and coupling efficiency, tenfold spiral is enough to match the radii of input OAM modes. Because there will be a strong transversal electric field component in the spiral slit, the initial radius of spiral must be large enough to make sure that there is no slit on the detecting plane. In this case, the longitudinal component of SPPs on the detecting plane is far stronger than the transversal component, so that the transversal component can be ignored. In the following, the Intensity distributions of SPPs denote the total Intensity of SPPs and the phase distributions denote the phase of longitudinal component.

**Figure 2:**
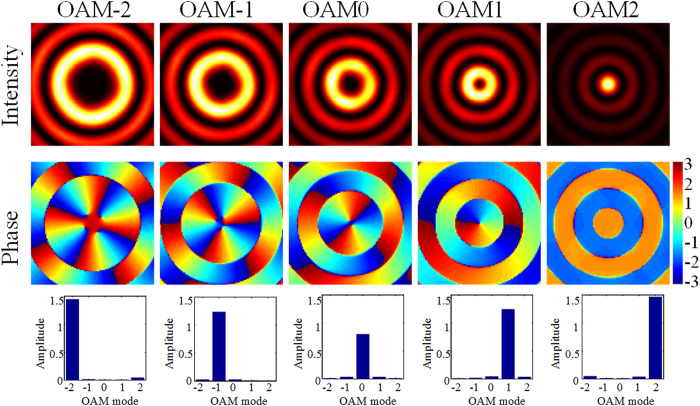
OAM-dependent plasmonic field distributions. First row, Intensity distributions. Middle row, phase distributions. Last row, retrieved amplitude distributions.

We use a three-dimensional finite difference time domain (FDTD) method to simulate the SPPs field. Without loss of generality, we assume that the input light contains five OAM modes whose TCs are equal to −2, −1, 0, 1, 2 (labeled as OAM-2, OAM1, OAM0, OAM1, OAM2). The input wavelength is 660 nm and the corresponding wavelength of the SPPs is calculated as 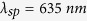. We set the initial values of 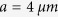, *m* = −1, *s* = −1 and *N* = 10. So the OAM mode with TC = *l* can be coupled to the SPPs in the form of 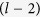-order Bessel functions. Figure 2 illustrates the simulated intensity distributions and phase distributions when different OAM modes with unit power illuminate the metallic film respectively. The size of pictures is 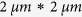. From Fig. 2, we can see that the OAM-2, OAM-1, OAM0, OAM1, OAM2 are coupled to the SPPs in the form of −4, −3, −2, −1, 0-order Bessel functions respectively. And the phase patterns signify that the SPPs have a −4, −3, −2, −1, 0-fold helical phase structure respectively. The simulated results consistently match the theoretical analysis shown by Eq. (2). Now, we decompose the intensity distributions of plasmonic field into Bessel functions by Eq. (5) and then determine the OAM distribution of input light. Note that the scheme can only get the relative phase of multiple OAM modes, so the phase retrieve is meaningless with only one OAM mode. The relative amplitude distributions are shown in the last row of Fig. 2. We can see that only the input OAM mode has a large amplitude and the amplitudes of other modes are close to null. And the RSR is always kept at a small value of about 0.01. It proves that the algorithm is accurate enough and the scheme can successfully detect the OAM mode when the input light contains only one OAM mode. In addition, we can find that the retrieved amplitude is inconsonant when changing the input OAM mode and the low order modes have lower coupling efficiency. It is caused by the mismatch between the geometric slits and the mode radii. In fact, the retrieved amplitudes (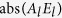) are exactly 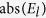 because all the 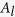 are previously set as the same value. Here, 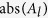 denotes the absolute value of 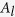.

**Figure 3:**
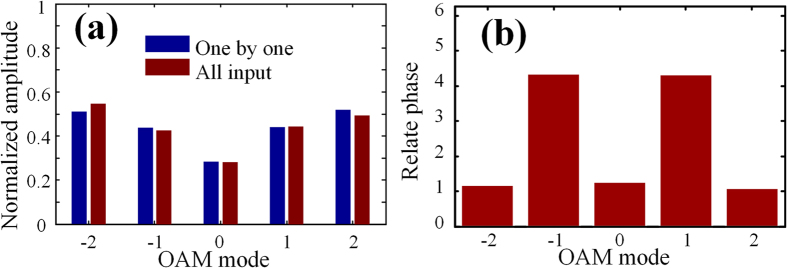
(**a**) Retrieved amplitude distribution and (**b**) phase distribution when OAM modes with the same power illuminate. The blue (red) bars denote the results by inputting the OAM mode with unit power one by one (all together).

In order to get the accurate OAM distribution of input light, we need firstly calculate the initial distribution of 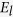 as a calibration. Although we can acquire the initial amplitude distribution of 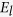 in Fig. 2, the phase distribution is absent. In fact, the distribution of 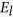 can be accurately calculated by Eq. (5) when all the OAM modes with the same power simultaneously illuminate. The calculated normalized amplitude distribution is presented as the red bars in Fig. 3(a). As a comparison, the blue bars denote the normalized results by illuminating the OAM mode with unit power one by one, i.e., the results originate from the last row of Fig. 2. One can see that the two calculated results are nearly identical. Owing to multiple OAM modes of input light, the relative phase distribution of 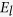 can also be acquired, as shown in Fig. 3(b). As expected, the conversion efficiency and phase shifts of OAM modes mapped to various Bessel functions are inconsistent. When detecting hybrid OAM modes, the distribution of 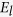 (both amplitude and phase) acts as the reference data for calibration.

**Figure 4:**
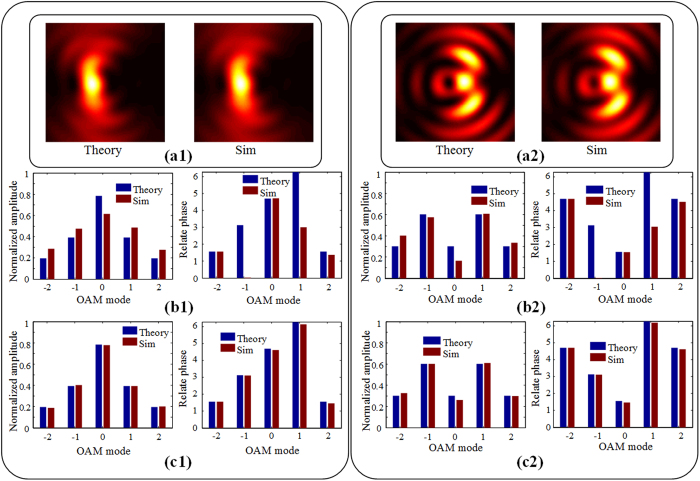
Results for hybrid OAM modes. (a1) The intensity distribution of SPPs. Retrieved complex amplitude distribution (b1) before calibration and (c1) after calibration. (a2–c2) Results of another example.

After getting the distribution of 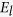, we can start to measure the OAM distribution of light. Two examples are given to prove the feasibility of the scheme. We assume the initial amplitude and phase distribution of the five OAM modes are set as (1, 2, 4, 2, 1) and (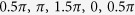) respectively. The theoretical intensity distribution of SPPs and simulated one are presented in Fig. 4(a1) when these OAM modes normally illuminate the metallic film. We can see that they match well. By decomposing the intensity pattern into the Bessel functions according to Eq. (5), a set of complex coefficients are obtained, the amplitude and phase distribution of the coefficients, which are related to the input OAM modes, are shown as the red bars in Fig. 4(b1). The blue bars exhibit the theoretical OAM distribution of input light. Here the RSR is lower than 0.002, but we can find that the measurement errors are a little bit large, especially in the phase distribution. The errors are caused by the different mapping coefficients from input OAM modes to SPPs. The accurate OAM distribution of input light can be calibrated through dividing the complex coefficients by the reference data in Fig. 3. The calibration results are depicted in Fig. 4(c1), who agree well with the theoretical ones. Another example is presented in Fig. 4(a2–c2) and the RSR is about 0.005. The initial amplitude and phase distribution of the five OAM modes are set as (1, 2, 1, 2, 1) and (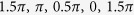) respectively. Similarly, both amplitude and phase have large measurement errors without calibration, and the calibrated results are precise enough. It proves that our scheme can well and truly detect the hybrid OAM modes.

## Discussion

In order to ensure the uniqueness of retrieved OAM distribution, the basic functions in Eq. (3) must have different intensity distributions, namely, the value of *m* + *s* + *l* should not change its sign for all the input OAM modes. The condition restricts the maximal order of discriminable OAM states. But it can be optimized by using PVL with higher order geometrical charge. Besides, we need first convert the input polarization state to the specified one (i.e., RCP or LCP) before measurement, because the premise of retrieve algorithm is illumination of circularly-polarization light. In fact, our scheme can also be applied to other polarization states provided that the basic functions in Eq. (3) were also revised accordingly. In addition, the measurement accuracy of OAM modes is mainly affected by the sampling points of detector and the measurement errors of plasmonic field distribution in actual measurement. To ensure the uniqueness of solution, the sampling points of detecting plane must be more than the number of OAM modes to be detected and the accuracy of solution will increase when increasing the sampling points. In the simulation, the sampling plane is set as 50 ∗ 50 pixels. Typically, the SPPs can be captured by the near-field scanning optical microscope (NSOM)^17^, but the NSOM collects much more transversal electric field component than longitudinal one which will affect the measurement accuracy. In fact, the SPPs can also be directly captured by a charge coupled device (CCD) when the CCD is placed in a plane exactly behind the plane of the PVL as close as possible^6^, or captured by the leakage radiation microscopy (LRM) which can collect the intrinsic radiation leaking through the gold film by using a high numerical aperture oil immersion objective^29^,^30^. These methods are more convenient and practical for our design.

In conclusion, we put forward an integrated scheme to retrieve the OAM distribution of light with PVL. The OAM modes are coupled to SPPs in the form of various Bessel functions respectively. The OAM distribution can be retrieved by decomposing the interference pattern of SPPs into these Bessel functions. Via calibration, our scheme can accurately measure the relative amplitude and relative phase of OAM modes simultaneously. It offers an integrated scheme to measure hybrid OAM states by one measurement.

## Methods

### Simulation method

We use a three-dimensional finite difference time domain (FDTD) method to simulate the SPPs field in gold-air interface to demonstrate the analysis model. The permittivity of the metal can be described by the Drude model 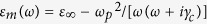, where 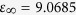 is the bulk permittivity at infinite frequency, 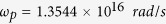 and 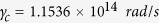 are the plasma frequency and the collision frequency, respectively^31^. For a gold-air interface, the wavelength of the SPPs is 635 nm when the wavelength of the incoming light is set as 660 nm. The dimensions of the total simulation area are 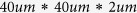. The spiral air-slits have a width of 200 nm and the gold film has a thickness of 200 nm. The metal film is deposited on the silica substrate. The monitor records the amplitude and phase of SPPs as shown in Fig. 2. The area of the monitor is 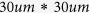. The effective detecting plane is a square with 50 ∗ 50 pixels and the size is 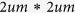.

## Additional Information

**How to cite this article**: Zhou, H. *et al.* Retrieving orbital angular momentum distribution of light with plasmonic vortex lens. *Sci. Rep.* **6**, 27265; doi: 10.1038/srep27265 (2016).
